# Challenges of the information age: the impact of false discovery on pathway identification

**DOI:** 10.1186/1756-0500-5-647

**Published:** 2012-11-21

**Authors:** Colin J Rog, Srinivasa C Chekuri, Mary E Edgerton

**Affiliations:** 1 M.D. Anderson Cancer Center, Department of Pathology, 1515 Holcombe Blvd, Houston, TX, 77030, USA

**Keywords:** Databases, Pathways, Genes, Networks, Bioinformatics, Cancer pathways

## Abstract

**Background:**

Pathways with members that have known relevance to a disease are used to support hypotheses generated from analyses of gene expression and proteomic studies. Using cancer as an example, the pitfalls of searching pathways databases as support for genes and proteins that could represent false discoveries are explored.

**Findings:**

The frequency with which networks could be generated from 100 instances each of randomly selected five and ten genes sets as input to MetaCore, a commercial pathways database, was measured. A PubMed search enumerated cancer-related literature published for any gene in the networks. Using three, two, and one maximum intervening step between input genes to populate the network, networks were generated with frequencies of 97%, 77%, and 7% using ten gene sets and 73%, 27%, and 1% using five gene sets. PubMed reported an average of 4225 cancer-related articles per network gene.

**Discussion:**

This can be attributed to the richly populated pathways databases and the interest in the molecular basis of cancer. As information sources become enriched, they are more likely to generate plausible mechanisms for false discoveries.

## Findings

As our knowledge about pathways increases, more genes are assigned to networks and the probability of generating a network from a randomly drawn set of genes is constantly increasing. Coincident with this is the fact that the number of publications relating genes to cancer is increasing; thus the probability of finding a paper on cancer that includes a gene listed as part of a “discovered” pathway is also increasing over time. The old adage, the more you know, the less you realize that you know can be modified to say that the more we know, the more likely we are to be misinformed by our knowledge. In conclusion,

1) Using pathways databases to support cancer related discoveries from data analysis of high throughput technologies can work to propagate false discoveries.

2) The more we know about pathways the more likely a set of genes or proteins under study, even if randomly selected, will be connected in some way in a pathways database.

3) While cancer was used for this case study, it is not the only disease that should be affected. The more we study the genetic and proteomic basis of a target disease, the more likely it is that a randomly generated network associated with a false discovery will be related in the literature to that target disease.

## Introduction

Modern research into the molecular biology of cancer can be traced back to the introduction of Knudson’s “two-hit” hypothesis based on his discovery of a second somatic mutation in tumors from patients with a germline retinoblastoma gene mutation [1]. Since then research into the molecular biology of cancer has evolved into an effort to integrate patterns from increasingly complex analysis of data. Twenty years after the “two-hit theory” was proposed, a study of colorectal cancers demonstrated a more complex scenario with most tumor samples demonstrating mutations in four to five genes [2]. A more recent study discovered that there could be up to 20 mutated gene that have a role in the evolution of a type of cancer [3]. The number of genes involved in tumorigenesis or tumor behavior becomes even larger when taking into account downstream effects that activate or disable a number of functional pathways, each of which may contain a network of many genes [4,5]. Thus, there can be many pathways that can be assigned a role for even a single type of cancer, and an even larger number of genes and/or proteins when the entire network or pathways is considered.

In addition to mutations affecting functional activity, copy number aberrations, microRNA and methylation have been identified as additional regulatory systems that, when defective, can contribute to cancer. The National Institute of Cancer has funded a large scale effort to generate a library of gene sequences, copy number aberrations, gene expression, methylation, and microRNA expression data for hundreds of tumor samples across multiple types of cancer. It has become clear that all of this information is starting to burden those who seek to use it to create new clinical care paradigms [6,7]. Thus, new approaches to storage, retrieval, and in particular analysis of this information in the context of functional pathways are necessary to understand the molecular basis of cancer [8].

Numerous approaches to integrating information from pathways databases with pattern analysis of the various types of –omics data have been demonstrated to be of utility in identifying pathways relevant to tumorigenesis and tumor progression, both from a pre-analysis perspective using, for example, gene enrichment analysis [9] or as a post-hoc strategy for interpreting the relationship between significant genes or proteins (e.g. [10]. MetaCore (distributed by GeneGO), Gene Ontology, Ingenuity Pathway Analysis (distributed by Ingenuity), MSigDB, Reactome, and the Kyoto Encyclopedia of Genes and Genomes are just some of the databases available that store information about relationships between genes and their protein products in a pathways context. MetaCore has in particular an attractive function using a graph theory algorithm to generate networks that connects genes and proteins submitted as input based on relations abstracted from the literature [11].

These databases are critical to analyzing –omics data in the context of pathway analysis. They are used to explore or substantiate pathways hypothesized from data analysis. However, the propagation of misinformation due to a non-zero false discovery rate in the gene sets selected as relevant has not been explored. As these databases become enriched with more information, the probability that any two input genes/proteins will have been documented as having a relationship within a network increases. Going one step further, if an association can be made between the members of the network and the clinical phenomenon or disease of interest can be made in the literature, then a hypothesis generated from a false discovery can be fortified by supporting information. This problem is potentially very meaningful in diseases that are well studied, such as cancer. Since 1960 there has been an exponential increase in the number of publications that reference the term “cancer” in PubMed; within the last year alone, there were nearly 50,000 articles.

Using cancer then as a case in point, this report demonstrates the potential pitfalls faced in using networks generated from a representative pathways database. Multiple sets of random solutions sets of genes are created and submitted to MetaCore to see if they can be linked together into functional networks. Members of the extended network returned from MetaCore are searched in PubMed for cancer relevance. The results indicate the ease with which false discoveries can be supported by bioinformatics, resulting in hypothesized mechanisms that are not truly supported by the data.

## Results

**Figure 1:**
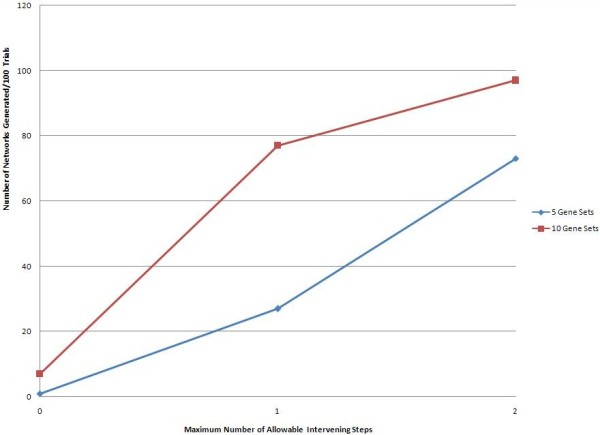
**MetaCore results.** Networks generated (y-axis) vs. maximum intervening edges allowed in MetaCore’s “Shortest Path” algorithm (x-axis).

**Figure 2:**
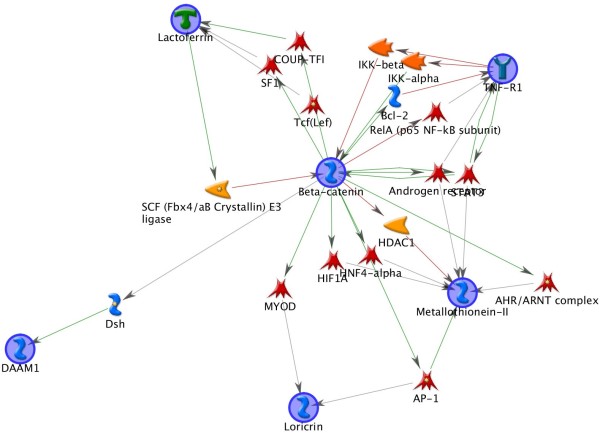
**Representative pathway.** This pathway was generated with ten input nodes and allowed for up to two edges between input nodes. The input nodes that are members of the pathway are encircled in the figure.

Using three, two, and one maximum intervening step between input genes to populate the network, at least one network was generated with frequencies of 97%, 77%, and 7% for the ten random gene sets and 73%, 27%, and 1% for the five random gene sets. As stated in the methods, these results were generated using 100 trials for each case, e.g. five genes with one intervening step allowed, and are displayed here in Figure 1. The positive relationship between the number of networks generated and the maximum steps allowed is to be expected, that is, the number of networks that are generated increases with an increasing number of allowed edges between input nodes. The increase is more dramatic for the ten gene sets, though both converge on 100%. Figure 2 depicts a representative pathway generated from ten input nodes and allowing up to two intervening edges.

**Figure 3:**
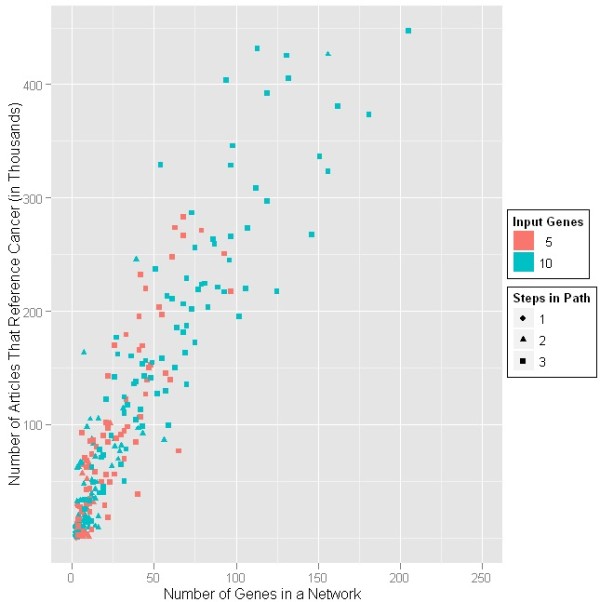
**PubMed results.** Articles retrieved from PubMed (y-axis) vs number of genes for the networks generated from MetaCore (x-axis).

As described in the methods, a query was constructed to search for publications that related any of the genes in the discovered network(s) for any one trial with cancer. This query was submitted to PubMed (see Figure 3). There is as expected a strong, positive relationship between the number of input nodes and the number of cancer articles. It is certain that this number includes more than articles elucidating a direct link between the gene(s) of interest and cancer, for example, a study that shows no relationship.

The different symbols and colors for the different classes of trials, where a class is defined by both the number of random genes and the maximum number of intervening edges allowed in the resulting network, in Figure 3 demonstrate the increase in the number of network nodes given more input genes (red symbols for ten random genes compared with black symbols for five random genes) and when more intervening edge are allowed (diamond symbols for three, triangles for two, and circles for one intervening edge). Some of the networks were associated with a very high number of articles. These are not shown in Figure 3 and were removed as “outliers”. These may be attributable to networks that contain highly studied oncogenic pathways such as src or p53. Excluding those data as potentially representing special cases, the slope of the curve is 2466 articles per input node.

## Discussion

These results demonstrate that pathways related to cancer can be readily generated using sets of genes selected randomly from a list of genes used in a standard Affymetrix microarray chip. Empirical testing clearly reveals that the pathways databases can function to amplify the misinformation resulting from false discovery by generating plausible mechanisms to support the results. In addition to obtaining realistic pathways, the support from literature associated with cancer enhances the potential for propagation of misinformation. As knowledge in the domain of pathways increases, more genes are assigned to networks and the probability of generating a network increases. Coincident with this is the fact that the number of publications relating genes to cancer is increasing, and the probability of finding a paper on cancer that includes a gene listed as part of the “discovered” pathway is also increasing over time. The old adage, the more you know, the less you realize that you know can be modified to say that the more we know, the more likely we are to be misinformed by our errors.

It has been pointed out that pathway modeling is one of the most active areas of data analysis for high throughput data [12]. Overfitting is a problem in statistical analysis of high throughput data as there are frequently fewer test subjects than measurements. In addition to this imbalance, the complex algorithms used in bioinformatics can adapt to random noise in data just as they do to actual patterns [13]. Even though techniques for filtering noise, determining significance, and accounting for large amounts of data have proven useful, even the smallest p-values used in studies can create substantial chance error simply because the genome is so large [14].

This means that false gene discovery combined with the expansion of information in pathways databases and literature search engines can lead to the propagation of misinformation. In other words, if input information is incorrect, there may be a relatively high chance of obtaining results that support these false results. Although we have used Metacore’s on-line tool to demonstrate the problems that can arise with pathways searches, we expect this problem to apply to other pathways databases as well, e.g. Ingenuity Path Designer Graphical Representation uses literature sources to generate edges between members of a pathway as does Metacore. Similarly Ariadne uses a database of relationships (ResNet Explore) that is used to generate pathways. Basically, the probability of detecting a network or pathway increases with the increasing size of the knowledge base of interactions in databases that are used to generate a pathway. This clearly presents a danger to research, especially to the field of personalized medicine as applied to cancer. While high throughput technologies have opened the door to novel discoveries for personalized medicine, they can also result in novel discoveries with plausible mechanisms that can be easily generated even if the genes of interest are randomly selected. Misleading hypotheses generated from analyses of high-throughput data are likely to be amplified by both pathway databases and reference libraries as investigators struggle to find significant results amongst all of this information.

Even though further text mining tools [15], and systematic use of keywords [16], might help filter unrelated retrievals, the problem of reviewing the literature in detail remains daunting considering the number of articles retrieved with the Boolean statements submitted. Given that cancer as a disease uses normal pathways even though it dysregulates their activity (overactive or underactive), then it is expected that many genes will have an association with cancer even if they are not causative. Therefore, a future focus is needed to minimize misleading results and to enrich for significant ones. Of course, better methods in the initial analysis to reduce false positive results help to reduce false pathway information, such as randomizing classes and repeating the data analysis. In addition, hypothesized pathways can be supported with some statistical analyses. For example, if “*k*” nodes of pathway “*A*” are in the input file to the pathways database, then how frequently would any combination of “*k*” nodes from pathway “*A*” be randomly selected from the complete data set (e.g. the probe set for the experiment). Also, percentage of a pathway’s membership that is selected from the initial data analysis as input is important. The higher the percentage of the total number of nodes in a pathway that are selected as statistically meaningful in the data analysis that generates the input nodes, then the more likely it is that the pathway is operating in the data. For example, if two of ten total nodes for pathway “*A*” are selected in the data analysis, the likelihood that the pathway is meaningful is less than if all ten of the nodes were selected in the initial data analysis.

Certainly, experiments are required to validate a hypothesized pathway and all pathway information should be treated with caution as a hypothesis until proven otherwise. To label a gene or pathway as definitively causal will require external validation in a laboratory setting, such as up- or down-regulation within engineered cell lines. In short, the finding of a network that integrates gene discovery into an acceptable hypothesis and relevant disease-related literature should not be considered strong supporting information.

## Methods

### Generating random lists of five and ten genes/proteins to represent false discoveries

One hundred sets of five random Unigene identifiers and one hundred sets of ten random Unigene identifiers were randomly selected using computer scripts with R library calls. The Unigene identifiers were selected using BrainArray’s (brainarray.mbni.med.umich.edu) Custom CDF file, Unigene Version 13.0.0, and applied to the Affymetrix HT HGU 133A platform as a representative list of genes used in expression microarray experiments. The complete set of Unigene identifiers contained 12001 entries, some of which were repeated, representing replicated probes in the chip. Gene sets were selected without replacement using the following R v2.11.1 scripts [17,18].

Gene names[sample(12001,5,replace = FALSE)] (select 5 out of 12001 gene names without replacement).

Gene names[sample(12001,10,replace = FALSE)] (select 10 out of 12001 gene names without replacement).

### Meta core pathway analysis

MetaCore is a pathway database that includes over 200,000 frequently updated protein-protein and protein-small molecule interactions that have been extracted from literature resources by experts. It allows the use of many different algorithms to analyze gene lists and handles large amounts of data very well [11].

MetaCore’s on-line tool that employ’s Dijkstra’s shortest pathway algorithm was used to measure the frequency with which the five and ten randomly selected Unigene identifiers could be mapped onto a set of genes that could generate pathways (“shortest pathway” option in “Build Network” menu). Notably, occasionally the Unigene identifiers were not mapped onto corresponding objects in MetaCore; other times one Unigene identifier mapped to more than one object in MetaCore. We excluded anything except known synonyms for the gene associated with the Unigene identifier. Thus, the results were interpreted as obtained from 5 randomly generated Unigene identifiers that potentially mapped to more or less than 5 MetaCore objects.

Networks were generated using Dijkstra’s algorithm. Both open and closed networks are allowed. These are drawn with single-step interactions (“edges”) connecting any two genes/proteins in the network [8]. Input options include the maximum number of intervening edges in the path between any two of the input objects, called “root objects” in MetaCore. Inputs of one to three intervening edges were compared for both the five and ten gene sets as input. One hundred (100) trials were performed for each of the five and ten gene sets and with each of one, two and three intervening steps for a total of 600 trials. The frequencies with which networks were generated were recorded. Canonical pathways were allowed; information was restricted to the Homo sapiens species.

### Pubmed literature search

To get a better idea of the massive amounts of information that could be garnered from the information provided by each network, an automated literature search was performed on PubMed, a search engine for biomedical literature that was developed by the National Center for Biotechnology Information [15]. A Boolean search term was constructed with an “AND” operator joining the term “cancer” to a list of the genes returned as nodes in the networks. The latter were joined by an “OR” operator. This was submitted in PubMed query format and was equivalent to manually entering:

“CANCER” AND “gene1” OR “gene2” OR “gene3” … “geneN” (where N represents the total number of genes in the network for a particular trial).

This search yielded all of the articles that included the term “cancer” and any of the genes of interest found in a particular network. No time limits or other constraints were placed upon the search.

## Competing interests

The authors declare that they have no competing interests.

## Authors’ contributions

Colin Rog completed this work as part of a summer internship in bioinformatics with Dr. Edgerton’s group. Mr. Rog carried out the work and completed the first draft of the manuscript. Srinivasa Chekuri, MD, MS developed scripts pertaining to the work and contributed to data analysis. Mary E. Edgerton, MD, PhD conceived of the study, supervised the research tasks, reviewed the data, and completed the final version for submission to the journal. All authors read and approved the final manuscript.

## Authors’ information

Colin Rog is an undergraduate student studying chemistry at Duke University. He completed this work as part of a summer internship in bioinformatics with Dr. Edgerton’s group.

Srinivasa Chekuri, MD, MS assisted with this work while studying for an MS in Biomedical Informatics at the University of Texas Health Science Center at Houston. Dr. Chekuri is current a resident in pathology at the University of Mississippi Medical Center.

Mary E. Edgerton, MD, PhD is a pathology informatician who has worked in the field of data mining for pathways relevant to clinical outcomes for patients with cancer. She is currently a collaborator on The Cancer Genome Atlas Genome Data Analysis Center grant awarded to John Weinstein, MD and Gordon Mills, MD at UT MD Anderson Cancer Center.
